# Gestational Trophoblastic Disease in Bahrain: Prevalence, Risk Factors, and Outcomes

**DOI:** 10.7759/cureus.89233

**Published:** 2025-08-01

**Authors:** Abrar M Alnasheet, Naeema Mahmood

**Affiliations:** 1 Obstetrics and Gynecology, Salmaniya Medical Complex, Manama, BHR

**Keywords:** choriocarcinoma, complete hydatidiform mole, gestational trophoblastic disease (gtd), gestational trophoblastic tumor, hydatidiform mole, invasive mole, molar pregnancy, partial hydatidiform mole

## Abstract

Background

Gestational trophoblastic disease (GTD) refers to a group of conditions linked to abnormal growth of trophoblastic tissue following conception. Although uncommon, early detection of GTD is vital due to the potential for progression and serious complications. The prevalence and presentation of GTD vary by region. In Bahrain, there are limited data on GTD, its risk factors, and clinical outcomes within the Bahraini population.

Objectives

To investigate the frequency, associated risk factors, and clinical outcomes of GTD among women in the Kingdom of Bahrain and to compare the findings with data reported in regional and international literature.

Methods

This study followed a retrospective cohort analysis. The study included all women with a confirmed diagnosis of GTD at the Salmaniya Medical Complex (SMC) between January 2018 and October 2023 (N=112). Data were collected by reviewing individual patients’ files and direct communication with patients via phone to complete any missing data. Collected data included maternal demographics, risk factors, histological diagnosis, follow-up period, and the need for chemotherapy.

Results

The mean age of included patients was 30.7±8.5 years, while the median and interquartile range for gravidity and parity were 2 (2.5) and 1 (2), respectively. Complete hydatidiform moles, which are diploid and composed entirely of paternal genetic material, were more prevalent in our cohort (71.4%). In comparison, partial hydatidiform moles, which are typically triploid and contain both maternal and paternal genetic material, accounted for 28.6%. Gestational trophoblastic neoplasia (GTN) developed in seven patients (6.4%), with one case presenting with lung metastasis. Possible risk factors included age, consanguinity, and blood groups (O+ and B+). For β-hCG to reach zero, complete moles, on average, took slightly longer to normalize at 54.6 days compared to 48.3 days for partial moles.

Conclusion

Our findings can serve as a foundation for developing targeted initiatives to improve the early identification and management of GTD. We recommend conducting future prospective studies that include data from private healthcare institutions and establishing a regional registry. This would help achieve a larger sample size and allow for a more comprehensive assessment of potential risk factors and clinical outcomes associated with GTD.

## Introduction

Based on the World Health Organization classification, gestational trophoblastic disease (GTD) represents a diverse collection of conditions linked to pregnancy, marked by the excessive growth of tissue originating from the placenta after abnormal fertilization. From a clinical-pathological point of view, GTD constitutes a group of tumors with an abnormal trophoblastic proliferation with premalignant and malignant forms [1].

Histologically, it is divided into hydatidiform moles (with villi) or other trophoblastic neoplasms (without villi). The premalignant forms of GTD include complete and partial hydatidiform moles (PHMs), which together account for approximately 80% of all GTD cases. Complete hydatidiform moles (CHMs) have a higher risk of progression to gestational trophoblastic neoplasia (GTN) compared to PHMs. On the other hand, the malignant variants can be either nonmetastatic or metastatic, which include invasive mole (15% of cases), choriocarcinoma (a rare form that makes up about 5% of cases), placental-site trophoblastic tumor (extremely rare), and epithelioid trophoblastic tumor (even rarer), collectively called GTN [2,3].

Hydatidiform moles represent the most prevalent form of GTD. Their incidence rates vary globally, with European reports indicating 0.5-3.0 cases per 1,000 pregnancies. In Asia, occurrences are more frequent, with figures spanning from 1 to 10 cases per 1,000 pregnancies [2]. Nutritional and hereditary factors might also play a role in these variations [4].

The CHM is of an androgenetic origin with a diploid chromosomal pattern, arising from a single sperm fertilizing an empty ovum, after which paternal chromosomes duplicate or two sperms fertilize a single ovum, with subsequent loss of maternal chromosomes. This leads to a diploid composition of exclusively paternal DNA with no fetal tissues [4]. On the other hand, the PHM typically arises when two sperms fertilize a normal egg, often resulting in a triploid set of chromosomes. Unlike CHM, the PHM may include some fetal tissues or fetal red blood cells [4].

GTNs stand out in cancers as they genetically stem from fetal tissues, namely, the trophoblastic cells of the placenta, and thus act as semi-allogeneic grafts within the patient. Hydatidiform moles, particularly invasive moles, are often medically regarded as a form of GTN due to their potential for local invasion and distant metastasis. Thus, biologically, they are characterized by abnormally developed placental tissues, not genuine neoplastic growths [4].

GTN can arise following any type of pregnancy, including molar pregnancy, miscarriage, ectopic pregnancy, or even term delivery. These malignancies are highly responsive to treatment and are considered highly curable, especially when diagnosed early. Conversely, placental site trophoblastic tumors and epithelioid trophoblastic tumors typically present with lower β-HCG levels and appear after a more extended period after pregnancy. Moreover, the occurrence of these conditions among young women who are of childbearing age renders them a significant concern for society [5-10].

Several risk factors have been associated with developing GTD, with maternal age being one of the most recognized. Both women under 20 and those over 40 appear to be at higher risk. Although factors such as declining birth rates and oral contraceptive use are not widely cited in the literature as established risks, they were included in our analysis to investigate any potential correlations within our population. On the other hand, the evidence linking GTD to lifestyle and environmental influences, such as smoking, alcohol consumption, dietary patterns, socio-economic status, and herbicide exposure, remains limited and inconclusive [5].

Close monitoring of beta-human chorionic gonadotropin (β-hCG) levels is essential for all patients diagnosed with GTD, and it is advisable to prevent pregnancy during this monitoring phase. Women with partial or CHM typically achieve resolution through surgery. However, up to 20% of women with CHM may experience persistent molar tissue, which can progress into an invasive mole. In rarer circumstances, this could further advance to choriocarcinoma. Both conditions necessitate additional medical intervention [6]. However, since the prevalence and presentation of GTD may vary by region, it is essential to develop an accurate and up-to-date national surveillance system, since underestimation of the magnitude and outcome of GTD may occur [11,12].

Our primary objective is to assess the magnitude and consequences of GTD among pregnant women treated at Salmaniya Medical Complex (SMC). Moreover, this study intended to identify the possible contributing risk factors for GTD and determine the minimum follow-up duration necessary for normalizing β-hCG levels and the consequences of the disease in our study population.

## Materials and methods

Study population

This study included all women who underwent suction evacuation and were diagnosed with GTD during the period from January 2018 to October 2023.

Study design and setting

We conducted a retrospective cohort study at SMC, one of the main tertiary-care referral centers in Bahrain. It provides comprehensive gynecological, surgical, and histopathological services.

Inclusion and exclusion criteria

Inclusion criteria were women with histopathologically confirmed GTD (complete or partial hydatidiform mole, invasive mole, or choriocarcinoma) who underwent suction evacuation and were followed at SMC.

Exclusion criteria included women who underwent evacuation for other causes without histological confirmation of GTD, those with incomplete or inaccessible records, and those who were lost to follow-up without sufficient clinical or histological data.

Data collection process

Data were extracted from the electronic medical records system (I-SEHA), the gynecology operating theater logbooks, and histopathology laboratory reports. To address missing or incomplete data, patients were contacted by phone using the contact information registered in their files. A standardized set of questions was used to collect missing information related to reproductive history, lifestyle risk factors, and post-treatment follow-up. Up to three contact attempts were made per patient. If no response was obtained after three attempts, the patient was considered lost to follow-up. Interviews were conducted by trained medical staff familiar with GTD management to ensure consistency and accuracy.

Data collection instrument

A structured data collection sheet was developed based on a review of published GTD literature and the study objectives. The form was pilot-tested on a sample of 10 patient records to ensure that it was comprehensive and practical for both chart review and patient interviews. Necessary adjustments were made to improve clarity and ensure consistency in data abstraction.

The data collection sheet included the following data: (1) maternal demographics and risk factors for GTD (age, gravidity, parity, gestational age, previous miscarriages, prior molar pregnancy, current smoking status, coffee consumption, blood group, and family history of GTD); (2) histopathological features and types of GTD, and the total number of deliveries during the study period; (3) blood level of β-hCG at the time of diagnosis and during follow-up periods; and (4) received chemotherapy (if used)

Statistical analysis

Data analysis was performed using the Statistical Product and Service Solutions (SPSS, version 27; IBM SPSS Statistics for Windows, Armonk, NY). Descriptive statistics were used to summarize demographic and clinical characteristics. Means and standard deviations, in addition to median and interquartile range (IQR), were reported for quantitative variables and frequencies with percentages for categorical variables. The estimated prevalence of GTD was calculated relative to the total annual numbers of live births during the study period (2018-2023), as the live birth statistics were the most consistently available reference for estimating prevalence.

Independent sample t-tests were used to compare continuous variables (e.g., age, β-hCG levels) between subgroups such as complete and partial moles. Moreover, multivariate binary logistic regression was applied to identify significant risk factors for GTD. A p-value of <0.05 was considered statistically significant.

Ethical approval for this study was obtained from the SMC Research Ethics Committee (Reference No. 58030623).

## Results

Clinical presentation

Sonographic findings in almost all cases of CHM showed the classic “snowstorm” appearance, while PHM typically presented as cystic changes within the placenta, often with a coexisting fetus or intrauterine gestational sac. In addition to ultrasound features, markedly elevated serum β-hCG levels, often accompanied by suppressed thyroid-stimulating hormone (TSH) levels due to β-hCG’s thyrotropic activity, further increased clinical suspicion of molar pregnancy. Based on these findings, patients were scheduled for suction evacuation. A total of 112 patients were subsequently confirmed to have molar pregnancy through histopathological examination following the procedure.

**Figure 1:**
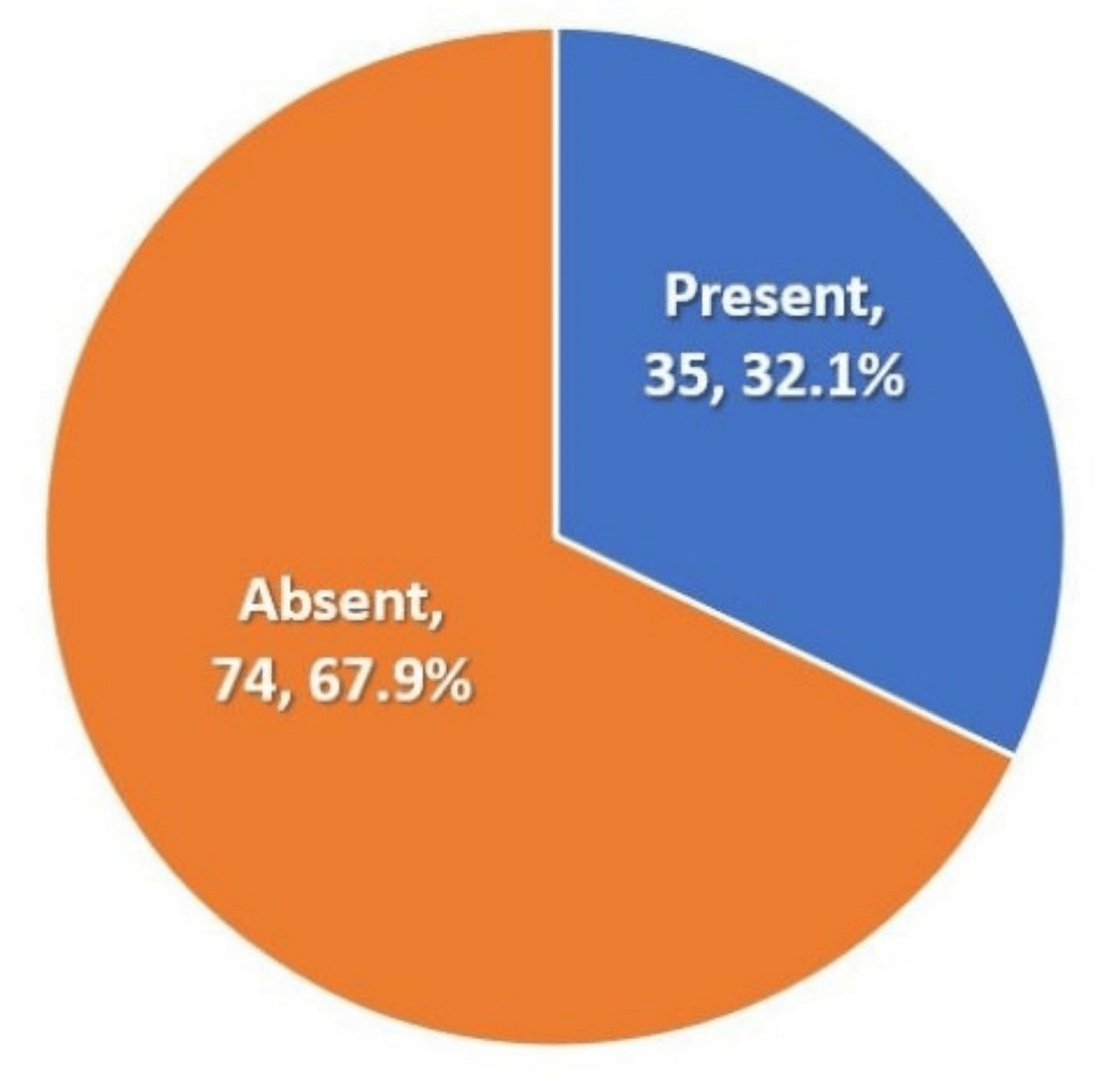
Hyperthyroidism among cases with gestational trophoblastic diseases

As a part of the hospital protocol, baseline chest X-rays and TSH levels were taken. All patients in our study had unremarkable chest X-rays. For TSH, the mean±standard deviation was 1.27 ± 2.04 mIU/L. The vast majority had low TSH levels and transient hyperthyroidism (Table 1, Figure 1).

**Table 1:** Patients’ TSH levels and hyperthyroidism TSH: thyroid-stimulating hormone

TSH Levels	No. of cases	%
Low (<0.55 mIU/L)	41	36.6
Normal (0.55–4.78 mIU/L)	68	60.7
High (>4.78 mIU/L)	3	2.7
Mean ± SD	1.27 ± 2.04 mIU/L	

Part 1: Estimated prevalence of GTD

**Figure 2:**
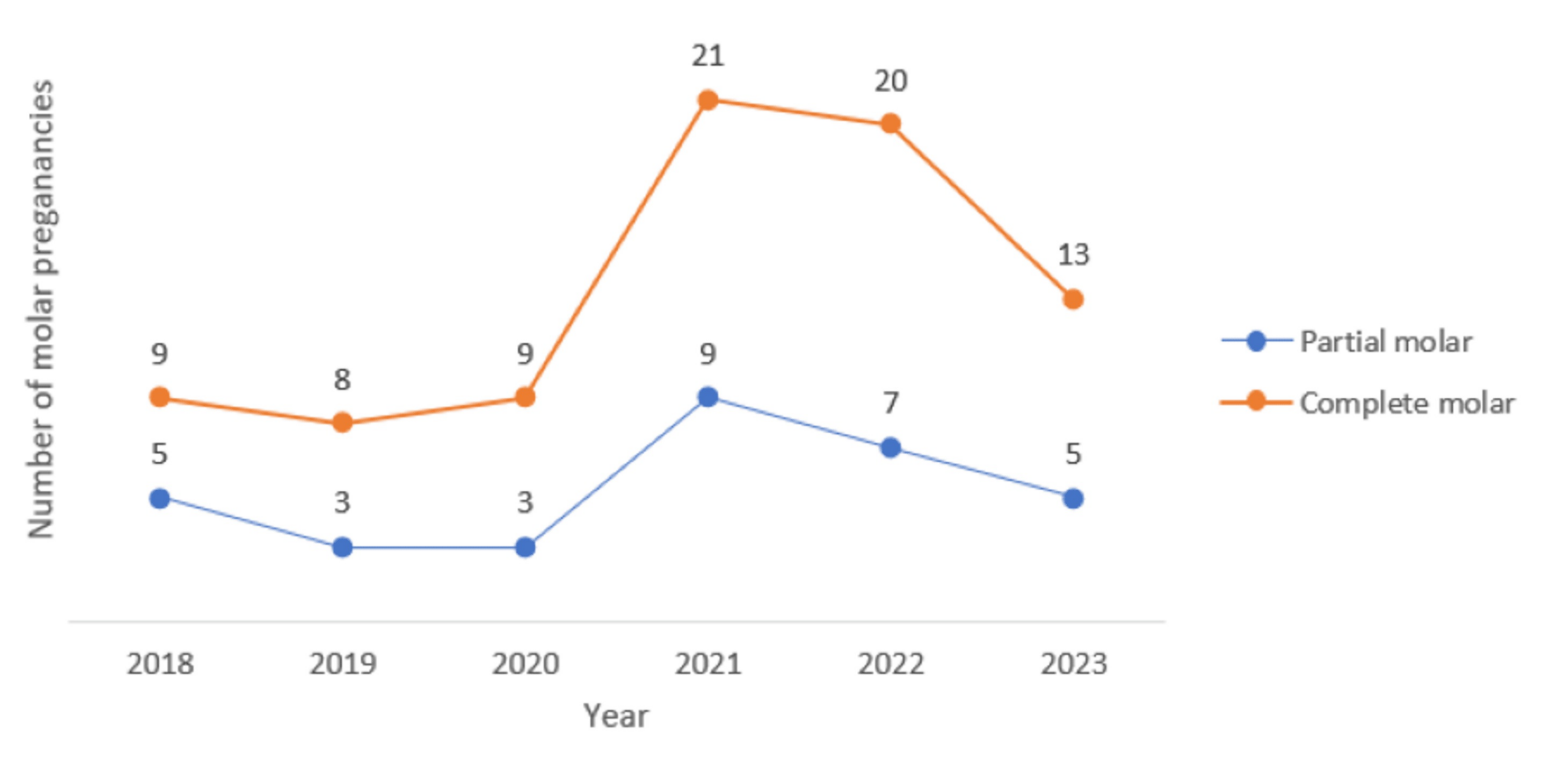
Types of molar pregnancies as number of cases from 2018 to 2023

All hospital files of patients admitted to SMC between 2018 and 2023 who were suspected to have GTD were examined (N = 198). However, only women who underwent suction evacuation and were diagnosed with GTD were included (n=112), while 86 women who proved not to have molar pregnancy were excluded. The lowest number of GTD cases was observed during 2018 and 2019 (14 cases and 11 cases, respectively), with estimated prevalence rates of 0.22% and 0.21%, respectively. On the other hand, the highest number of GTD cases (30 cases, estimated prevalence rate of 0.59%) was diagnosed in 2021 (Table 2). Out of 112 GTD patients, 80 had complete mole, and 32 had partial mole, as shown in Table 3 and Figure 2.

**Table 2:** Estimated prevalence of molar pregnancy, as a percentage of total live births from 2018 to 2023

Year	No. of examined files	Excluded cases	Included cases	Livebirths	Prevalence (%)	Ratio
2018	28	14	14	6392	0.22	1:457
2019	23	12	11	5243	0.21	1:477
2020	25	13	12	4497	0.27	1:375
2021	49	19	30	5077	0.59	1:169
2022	48	21	27	8276	0.33	1:307
2023	25	7	18	6723	0.27	1:374
2018-2023	198	86	112	36,208	0.31	1:323

**Table 3:** Prevalence of the types of molar pregnancy (as number of cases) from 2018 to 2023

Year	Partial hydatidiform mole		Complete hydatidiform mole	
	No.	%	No.	%
2018	5	35.7	9	64.3
2019	3	27.3	8	72.7
2020	3	25	9	75
2021	9	30	21	70

Part 2: Participants’ demography and obstetric profile

In this study, we included women who presented with vaginal bleeding and had suspicious ultrasound findings suggesting partial or complete mole, such as a snowstorm appearance or a placenta with a cystic appearance. Moreover, we studied 112 women who had suction evacuation and were found to have a confirmed diagnosis of molar pregnancy by histopathology report.

The majority of cases involved Bahraini women, accounting for 77 patients (68.8%), compared to 35 non-Bahraini patients (31.3%). Most patients were between 25 and 29 years old (35 patients, 31.3%) or 30-34 years old (27 patients, 24.1%), while 18 patients (16.1%) were aged 40 years or above. Regarding parity, most patients were parous, with 68 (60.7%) having a parity of 1-4 and seven (6.3%) with parity of 5 or more; nulliparous women accounted for 37 cases (33.0%). Additionally, 16 patients (14.3%) reported a history of one to four abortions. The majority of cases (60 patients, 53.6%) were diagnosed at a gestational age of 9-12 weeks (Table 4).

**Table 4:** Patients’ demographic and obstetric characteristics

Characteristics	No. of cases	%
Age (in years)		
<20	2	1.8
20-24	23	20.5
25-29	35	31.3
30-34	27	24.1
35-39	7	6.3
>40 years	18	16.1
Mean ± SD	30.7 ± 8.6 years	
Nationality		
Bahraini	77	68.8
Non-Bahraini	35	31.3
Gravidity		
G1	29	25.9
G2-G4	57	50.9
G5+	26	23.2
Median (Interquartile range)	2 (2.5)	
Parity		
0	37	33.0
1-4	68	60.7
5+	7	6.3
Median (Interquartile range)	1 (2)	
Abortion		
0	84	75.0
1-2	16	14.3
2+	12	10.7
Median (Interquartile range)	0 (0.8)	
Gestational age (in weeks)		
4-8	30	26.8
9-12	60	53.6
13-16	17	15.2
17-20	5	4.5
Mean ± SD	10.5 ± 3.3 week	

Part 3: Possible risk factors

The vast majority (109 patients, 97.3%) did not have a previous molar pregnancy. However, a positive family history of molar pregnancy was present in nine patients (8%). Consanguinity was present in over one-third of the patients (44 patients, 39.3%). Moreover, we wished to study whether metabolic syndrome is a risk factor for molar pregnancy.

Assessment of body mass index (BMI) was not feasible, as height measurements were not consistently recorded for all admitted patients. However, available weight data indicated that the majority of patients (n = 49, 43.8%) fell within the 51-70 kg weight range.

The majority of patients did not report any comorbidities, such as diabetes, hypertension, and dyslipidemia; only 32 patients reported having comorbidities (28.6%).

About one-third of patients regularly consumed caffeine (38 patients, 33.9%), with a daily intake of two cups of coffee or more. Nevertheless, the majority did not smoke themselves (91 patients, 94.8%), but more than one-third of patients had partners who were smokers (43 partners, 38.4%). Other factors studied were blood types and folic acid consumption. The most common blood types were O+ (57 patients, 50.9%) and B+ (21 patients, 18.8%). Taking folic acid was reported by only 13 patients (11.6%) (Table 5).

**Table 5:** Patients’ possible risk factors ‡ Comorbidities include diabetes, hypertension, and dyslipidemia.

Possible risk factors	Number of cases	%
Weight (in kg)		
<50	17	15.2
51-70	49	43.8
71-90	23	20.5
>90	6	5.4
Associated comorbidities‡	32	28.6
Smoking status		
Nonsmoker	91	94.8
Smoker	21	5.2
Husband’s smoking status		
Nonsmoker	69	61.6%
Smoker	43	38.4%
Daily intake of caffeine		
No	74	66.1
Yes	38	33.9
Previous molar pregnancy		
No	109	97.3
Yes	3	2.7
Family history of molar pregnancy		
No	103	91.0
Yes	9	8.0
Consanguinity		
No	68	60.7
Yes	44	39.3
Blood group type		
A+	25	22.3
AB+	6	5.4
B-	1	0.9
B+	21	18.8
O-	2	1.8
O+	57	50.9
Folic acid intake during pregnancy		
No	99	88.4
Yes	13	11.6

Part 4: β-hCG levels, follow-ups, and outcome

The mean β-hCG level was higher for complete moles (260, 291.6 mIU/mL), compared to partial moles (157,122.5 mIU/mL). Additionally, in our sample, there was no statistically significant difference (p > 0.05) in the mean highest β-hCG levels between those with and without a tumor. Those with a tumor had a mean of Β-hCG (236,073.8 mIU/mL) than those without (2,222,326.8 mIU/mL).

Patients were monitored with weekly serum β-hCG measurements to evaluate disease regression and identify potential cases of persistent trophoblastic disease. On average, β-hCG levels normalized within 7.5 ± 3.4 weeks. Approximately 51.5% of patients achieved undetectable β-hCG levels within one to six weeks, while the remainder required 7-20 weeks. There was no statistically significant difference in the mean time to β-hCG normalization between CHM and PHM (p = 0.436). The mean duration for β-hCG resolution was 7.8 weeks for complete moles and 6.9 weeks for partial moles (Table 6).

**Table 6:** Patients' follow-up with the β-hCG level

Possible risk factors	No. of cases	%
Readmission		
No	93	83.0
Yes	19	17.0
β-hCG level reaching zero (in weeks)	No. of cases	Mean ± SD
Partial mole	32	6.9 ± 2.5
Complete mole	80	7.8 ± 3.8
t-value, df (p-value)	1.24, 110 (0.219)	

A total of 20 patients were readmitted following their initial treatment due to persistently elevated β-hCG levels (p < 0.0001), necessitating further evaluation. These patients either underwent a second suction evacuation or were assessed with computed tomography (CT) scans to rule out invasive mole or metastatic disease. Only one patient required a hysterectomy due to uncontrolled hemorrhage during the evacuation procedure (Table 6).

Gestational trophoblastic neoplasia (GTN)

A small percentage of patients with molar pregnancy (seven cases, 6.4%) progressed to a tumor, and only one of those had lung metastasis. Of these patients, five were CHM, and two were PHM. PHM progressed to invasive mole in one case, and the other case progressed to choriocarcinoma. There was a 100% success rate in recovery from the neoplasm with a single chemotherapy agent (methotrexate) (Table 7).

**Table 7:** Number of patients with GTN GTN: gestational trophoblastic neoplasia

Outcome	No. of cases	%
Development of neoplasms			
No	105	95.6
Yes	7	6.4
Invasive mole	5	4.5
Choriocarcinoma	2	1.9
Metastasis		
No	6	5.4
Yes	1	0.9

To identify significant risk factors for PHM/CHM, binary logistic regression analysis (Table 8) revealed that a patient's nationality (being non-Bahraini) and obesity were significantly associated with having complete moles (p = 0.01 and p = 0.011, respectively).

**Table 8:** Multivariate binary logistic regression model for risk factors of gestational trophoblastic disease ‡ Statistically significant (p < 0.05) GTD: gestational trophoblastic disease

95% CI for EXP(B)							
Variables in the equation	B	SE	Wald	P-value	Exp (B)	Lower	Upper
Age groups	1.674	0.734	5.203	0.023	5.333	1.266	22.472
Nationality	-1.357	0.524	6.708	0.010‡	0.257	0.092	0.719
Gravidity	-0.547	0.327	2.804	0.094	0.578	0.305	1.098
Abortion	0.720	0.549	1.718	0.190	2.055	0.700	6.030
Parity	0.441	0.347	1.614	0.204	1.554	0.787	3.067
Consanguinity	0.379	0.574	0.436	0.509	1.461	0.474	4.500
Family history of GTD	1.618	0.983	2.709	0.100	5.045	0.734	34.659
Cigarette smoking	1.471	1.178	1.559	0.212	4.353	0.433	43.804
Caffeine intake	0.354	0.537	0.434	0.510	1.424	0.498	4.076
Weight category	-1.436	0.567	6.415	0.011‡	0.238	0.078	0.723
Constant	1.967	1.763	1.245	0.265	7.152		

## Discussion

This study included only histopathologically confirmed cases of molar pregnancy managed at SMC between January 2018 and October 2023. The predominance of CHM in our cohort aligns with findings from regional and global literature [6]. While the number of cases fluctuated slightly over the years, a general increase in the prevalence of GTD was observed.

During the study period, the prevalence of molar pregnancies ranged from 0.21% to 0.59% of total live births annually, with an overall prevalence of 1:323 live births (Table 2). These rates are comparable to those reported in Iraq (1:318) and Saudi Arabia (1:452) [11,13], while higher prevalence rates were documented in Oman (1:201) and Yemen (1:164) [6,14].

Notably, the highest number of both complete and partial moles was recorded in 2021. While no direct causal link has been established between COVID-19 and molar pregnancy, a 1.6-fold increase in case rates was observed when comparing the pre-pandemic period (2018-2019) to the pandemic/post-pandemic period (2020-2023). One possible explanation is the shift in healthcare access patterns during the pandemic, with fewer women attending private clinics and an increased reliance on government facilities such as our center, which remained operational for COVID-19-positive patients (Tables 2, 3). This increase in GTD prevalence during the COVID-19 pandemic may reflect healthcare system-related factors specific to the local setting.

Asian ancestry has been identified as an independent risk factor for GTD. Consequently, we focused our comparisons on studies conducted in Asian populations, where the prevalence of molar pregnancy is generally higher. Although the reasons for this increased susceptibility remain unclear, several studies have consistently reported elevated GTD rates among women of Asian descent [10].

In the present study, we identified 80 cases of CHM, 32 cases of PHM, five cases of invasive mole, and two cases of choriocarcinoma. This distribution is comparable to findings reported in Saudi Arabia and Iraq. In contrast, an Omani study reported a higher proportion of PHM than CHM, with 35 PHM cases and 28 CHM cases. Similarly, a study from Yemen documented 40 cases of PHM (50%) and 35 cases of CHM (43.75%) [6,11-14]. These differences may reflect variations in sample size, population characteristics, or diagnostic practices across settings.

Moreover, findings of the multivariate binary regression analysis indicated that being Bahraini was significantly associated with having a PHM. This finding is in accordance with that of Zakaria et al. [12], who noted that the incidence of GTD varies by region.

Most GTD cases in our cohort occurred in women aged 20-39 years, with the peak incidence in the 25-29 age group. Only two cases involved women under 20. These findings align with Omani data but differ from other international studies that report increased risk at maternal age extremes (<20 or ≥40 years) [15-18].

Other potential risk factors examined in this study included blood group, caffeine intake, smoking status, and secondhand smoke exposure. Currently, there is insufficient evidence in the literature to establish a clear association between smoking and increased risk of molar pregnancy. In our study, most women were non-smokers (94.8%), but 38.4% reported secondhand smoke exposure. A study from Yemen raised the possibility that partner use of qat (khat), which contains amphetamine-like compounds, may influence molar pregnancy risk, though a causal mechanism remains unclear [14].

The most common blood types among patients were O+ (50.9%) and B+ (18.8%), differing from prior studies that identified blood types A and AB as potential risk factors [15] (Table 5). The relevance of blood group as a risk factor remains controversial and may vary across populations.

Regarding BMI, binary logistic regression analysis revealed that obesity was a significant variable associated with having complete moles among our patients. However, to date, we have not identified studies specifically examining the association between obesity and the risk of molar pregnancy. This gap in the literature underscores the need for future prospective studies with standardized anthropometric assessments to explore the potential association between body weight and GTD risk.

With respect to parity, the majority of affected women were parous, particularly those with parity of 1-4, while 33% were nulliparous. This trend is consistent with the Omani study [19,20], though a study from Mexico found no significant association between parity and GTD risk [21].

Seven women in our cohort progressed to GTN, of whom six received single-agent chemotherapy (methotrexate with leucovorin) and responded well, requiring no more than five cycles. This aligns with global literature supporting the effectiveness of single-agent therapy for low-risk GTN [22].

A majority of cases in our study (75%) had no prior history of abortion, while 10.7% had experienced two or more previous abortions. In contrast, a study from India identified abortion as the most common antecedent event before GTD diagnosis. This may be attributed to the high rate of unregulated induced terminations in some settings, where molar pregnancies may not be properly evaluated histologically. As a result, many cases could remain undiagnosed, potentially underestimating the true prevalence of GTD in such populations [23].

After surgery, patients were monitored with weekly serum β-hCG measurements until their levels returned to normal. Once β-hCG became undetectable, monthly monitoring continued for one year to ensure sustained remission. Although there was a slight difference in the average time required for β-hCG levels to normalize between partial and complete molar pregnancies, this difference was not statistically significant.

CHM cases, on average, took slightly longer to normalize at 54.6 days compared to 48.3 days for PHM. Research in Oman indicated that the average timeframe for β-hCG levels to return to normal was 64 days for CHM and 62 days for PHM, with no statistically significant difference observed. This finding aligns with other studies in which the average duration to achieve a negative β-hCG value was 56 days [24,25].

Approximately one-third of patients in our study presented with clinical or subclinical hyperthyroidism, a finding consistent with previous literature, particularly among cases of CHM. Hyperthyroidism in molar pregnancy is attributed to excessively elevated β-hCG levels, which can mimic TSH and stimulate thyroid function. Additionally, a variant known as molar thyrotrophic hormone, with a larger molecular size and prolonged activity, may contribute to this effect. In accordance with hospital protocol, all patients with suspected molar pregnancy underwent TSH testing, enabling timely detection and management. Although typically self-limited following molar evacuation, molar pregnancy-induced thyrotoxicosis can occasionally escalate into thyroid storm, a rare but life-threatening complication [26-28].

Limitations and strengths

As with any retrospective study, certain limitations should be considered. The use of medical records and patient phone interviews may introduce minor inconsistencies or recall bias, particularly in cases with incomplete follow-up. In addition, the single-center design may limit the generalizability of the findings, and the absence of a control group restricts comparative analysis. The small number of patients requiring chemotherapy also limits the ability to draw firm conclusions about treatment outcomes. Moreover, the study period coincided with the COVID-19 pandemic, which may have influenced referral patterns and access to healthcare. A modest increase in GTD prevalence during this time, along with a high proportion of consanguinity in our cohort, may reflect local demographic or system-level factors that could differ from other regional settings and warrant further exploration.

Nonetheless, the study includes one of the largest histologically confirmed GTD cohorts in Bahrain, spanning a six-year period. Standardized diagnostic and follow-up protocols, combined with comprehensive data collection, enhance the reliability of the findings. To our knowledge, this is the first study in Bahrain to explore GTD trends in detail and provides a valuable foundation for future research and the development of regionally relevant clinical guidelines.

## Conclusions

This study provides valuable insight into the clinical and histopathological profile of GTD in Bahrain, indicating that complete mole is the predominant subtype. Most cases occur among multiparous women of reproductive age. No strong associations were found with smoking, caffeine intake, or blood group, while secondhand smoke exposure and consanguinity emerged as potential areas for further investigation.

Women's obesity proved to be a significant factor related to the type of hydatidiform mole. Moreover, thyroid dysfunction is frequent among GTD cases, particularly in those with CHM, while PHM is more prevalent among Bahraini patients. Future studies incorporating prospective designs, multiple healthcare institutions, and larger sample sizes are recommended to improve generalizability and validate the associations observed in this cohort.
